# Comparison of viral phenotype and inflammatory biomarker responses in acute HIV-1 subtype A and C infections

**DOI:** 10.3389/fmicb.2025.1649731

**Published:** 2025-08-11

**Authors:** Samantha McInally, Daniel T. Claiborne, Elina El-Badry, Rui Xu, Qianhong Qin, Zachary Ende, Martin J. Deymier, Jake W. Rhodes, Jill Gilmour, William Kilembe, Etienne Karita, Susan A. Allen, Ling Yue, Eric Hunter

**Affiliations:** ^1^Emory Vaccine Center at Emory National Primate Research Center, Atlanta, GA, United States; ^2^Faculty of Medicine, Imperial College, London, United Kingdom; ^3^Centre for Family Health Research Zambia, Lusaka, Zambia; ^4^Centre for Family Health Research, Kigali, Rwanda; ^5^Department of Pathology and Laboratory Medicine, Emory University, Atlanta, GA, United States

**Keywords:** HIV-1 transmitted founder virus, subtype A, subtype C, replicative capacity, inflammatory chemokines/cytokines, HIV pathogenesis, disease progression, infectious molecular clones

## Abstract

**Introduction:**

HIV-1 subtype A and subtype C infections have different rates of clinical disease progression, with subtype C infected individuals in the IAVI Protocol C multisite acute infection cohort having a 60% faster CD4 loss compared to subtype A.

**Methods:**

In order to investigate whether differences were due to the phenotype of the transmitted founder virus (TFV), or inflammatory cytokines and chemokines, known to drive pathogenesis, we PCR amplified, sequenced and constructed infectious molecular HIV-1 clones from the plasma of 30 acutely infected individuals in Rwanda and Zambia. We next compared the inflammatory plasma cytokine/chemokine profiles of individuals pre- and post-the estimated date of infection of 20 Rwandan individuals infected with subtype A and 34 Zambians infected with subtype C HIV-1.

**Results:**

A comparison of the replicative capacity of 14 subtype A and 16 subtype C TFV showed that they had similar replicative capacity (RC) scores. Nevertheless, high TFV RC scores were linked to more rapid CD4^+^ T cell loss, and higher inflammatory cytokine levels irrespective of subtype. Multivariable analyses showed that individuals infected with subtype C exhibited a significant increase in the levels of eleven pro-inflammatory cytokines/chemokines after infection, while, in subtype A infections only six cytokines were significantly elevated postinfection. Despite these differences, at 3-months post infection, similar overall biomarker profiles were observed in individuals infected with subtype A or subtype C viruses, primarily due to higher pre-infection baseline biomarker levels in Rwanda. In the combined cohort, we found a highly significant association between faster CD4^+^ T cell decline and higher levels of ITAC (CXCL11), which in turn was linked to higher TFV RC.

**Discussion:**

Overall, the data presented here argue against TFV RC as the basis for different pathogenic outcomes in the subtypes A and C. Moreover, levels of inflammatory cytokines that might drive disease progression were similar during acute infection indicating that additional studies are required to understand the mechanism underlying differences in disease progression between the two subtypes. For both subtypes, high levels of ITAC during acute HIV-1 infection are linked to rapid disease progression.

## Introduction

Almost 40 years after its initial discovery, HIV-1 remains a major health crisis worldwide with over 39.9 million individuals infected in 2023 and 1.3 million new infections in 2018. While HIV-1 infections occur across the globe, the heaviest disease burden is present in sub-Saharan Africa, harboring over 2/3 of all HIV-1 cases (UNAIDS, 2023).

HIV-1 demonstrates a great potential for generating genetic diversity due to lack of proof-reading ability in the reverse transcriptase and high viral recombination rates (Hemelaar, 2012; Hemelaar et al., 2019). In order to organize the amount of genetic diversity, HIV-1 group M is divided into nine different subtypes, A–D, F–H, J, K, and L (Hemelaar, 2012; Hemelaar et al., 2019; Yamaguchi et al., 2020). Globally, the two most common subtypes observed are subtype C (47% of global infections) and A (10% of global infections); these two subtypes are also the most common subtypes observed in sub-Saharan Africa (Hemelaar et al., 2019).

Clinical differences can be observed in infections by the nine subtypes globally. Subtype D, a common subtype in sub-Saharan Africa, was found to be associated with increased mortality compared to subtype A and has a 4-fold higher rate of CD4^+^ T cell decline in the absence of anti-retroviral drugs compared to other subtypes (Easterbrook et al., 2010; Hemelaar, 2012). Individuals infected with subtype D have also been reported to have weaker humoral immune responses to HIV infections compared to individuals infected with other subtypes (Longosz et al., 2014).

While there are numerous studies documenting characteristics of subtype C infections, studies exploring the features of subtype A infections are less common. However, one of the early studies looking at subtype A infections found that female sex workers infected with non-subtype A viruses were 8 times more likely to develop AIDS compared to those infected with subtype A (Kanki et al., 1999).

A multi-site study of acute-early HIV infections, termed IAVI Protocol C, enrolled over 600 newly infected individuals, who were infected by subtype A, C, D and recombinant viruses, and who were followed for as long as 10 years with regular viral load and CD4^+^ T cell measurements (Landais et al., 2016; Price et al., 2020). Studies within Protocol C found that infections with subtype A and C viruses resulted in different clinical presentations in the infected patients. Individuals infected with subtype C were found to have a 60% faster disease progression compared to subtype A, based on viral load, CD4^+^ T cell decline, and time to AIDS (Amornkul et al., 2013). Moreover, in the IAVI Protocol C cohort, a higher proportion of viral controllers and long-term non-progressors were observed following subtype A infections, compared to individuals infected with subtype C in the absence of antiretroviral therapy (Price et al., 2019). In addition, in multivariate analyses viral control was shown to be independent of favorable HLA alleles (Prentice et al., 2014; Price et al., 2019). Finally, in HIV discordant couples, where one partner was infected with HIV, subtype C in Zambia was associated with higher post-counseling HIV-1 incidence compared to subtype A in Rwanda despite the same counseling and interventional approaches in each country (Kamali et al., 2015). Collectively, these data indicate that virus-specific characteristics may determine a less pathogenic outcome in subtype A infected individuals.

In this study, we sought to investigate possible causes for the observed clinical differences seen between HIV-1 subtype A and C infections and if these differences could be observed during acute HIV-1 infection. To address this question, we compared the replicative capacity of transmitted-founder viruses from each subtype, since we have shown previously that the trajectory of HIV pathogenesis is defined in part by this trait of the virus. In addition, because inflammation and inflammatory cytokines are linked to more rapid disease progression in HIV-1 infected individuals (Kuller et al., 2008; Prince et al., 2012; Claiborne et al., 2015), we examined the cytokine/chemokine profiles of individuals pre- and post-infection with either subtype A or C HIV-1 to see if there were any observable differences in the biomarker response in the acute phase of infection between subtypes.

## Results

### Replicative capacity scores are similar for subtype A and subtype C transmitted-founder viruses

**Figure 1:**
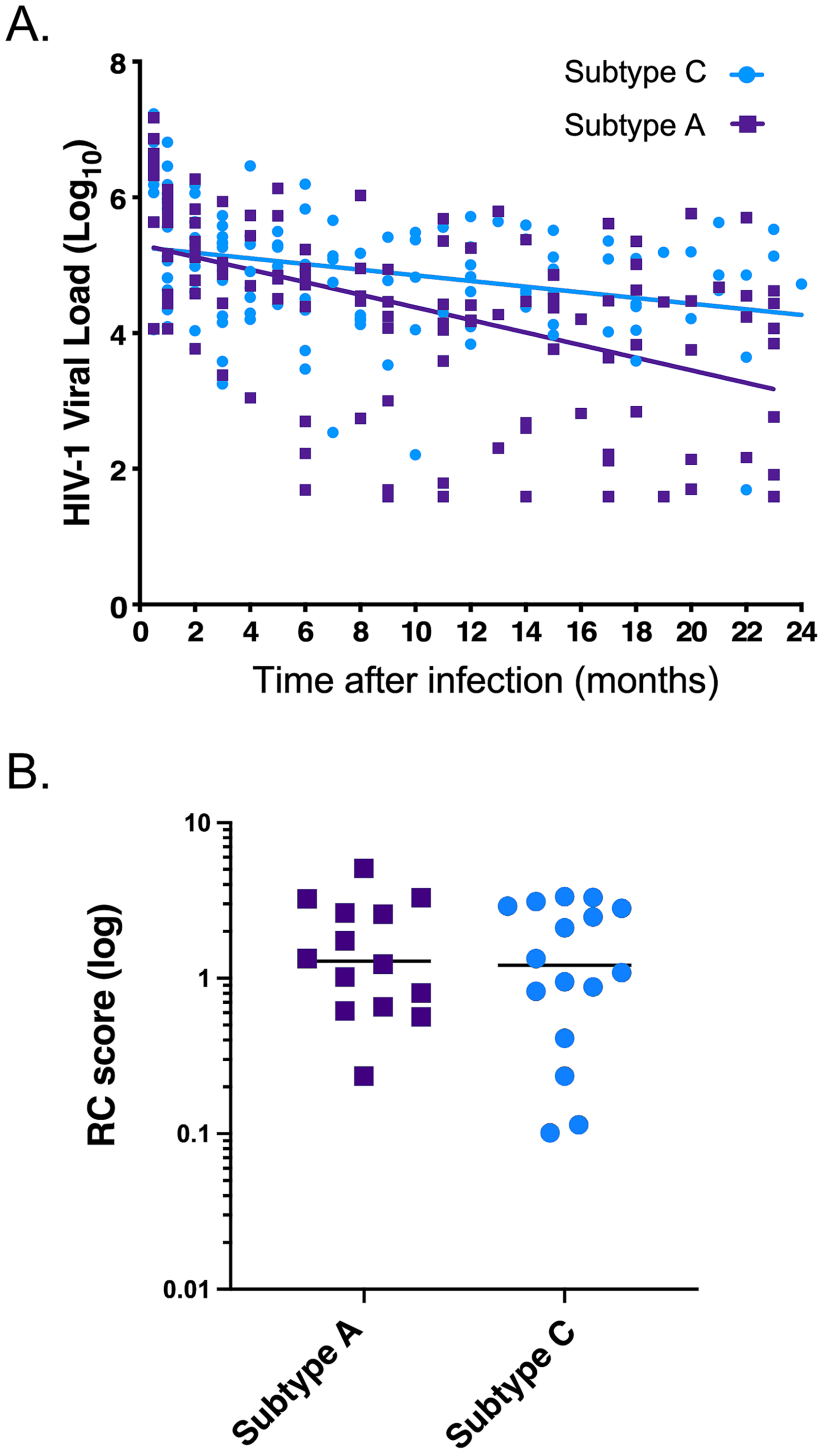
Comparison of viral phenotypes during acute infection of HIV-1 subtype A and C transmitted-founder (TF) viruses. **(A)** Viral loads over the first 2 years post infection of the individuals from which the IMCs were generated from plasma. Purple indicates subtype A viral loads (*n* = 14) and light blue indicates subtype C viral loads (*n* = 16). Lines show a linear regression analysis of the data as described in Methods, with a significant difference between the slopes (*p* = 0.0061). **(B)** Replicative capacity score of the subtype A and C TF viruses tested. Purple indicates subtype A RC scores, and light blue indicates subtype C RC scores. Mann–Whitney, two-tailed test (*p* = 0.821).

To investigate subtype differences in disease progression, we first compared the abilities of subtype A and subtype C transmitted-founder viruses (TFV) to replicate *in vitro* in activated PBMCs. We were able to construct authentic transmitted-founder virus infectious molecular clones (IMC) from 14 individuals acutely infected with HIV-1 subtype A and 16 individuals acutely infected with HIV-1 subtype C for this study. All samples used in the PCR amplification and cloning of TFV in this study were collected a median of less than 30 days post the estimated date of infection [(EDI)—Subtype A—Median 19 (range 10–73) and Subtype C—Median 25 (range 8–66)] as shown in Table 1. When we plotted the viral loads of these individuals over the first 2 years of infection, we found that individuals infected with subtype A HIV-1 exhibited better viral control compared to individuals infected with subtype C (Figure 1A). This result is consistent with findings obtained in an analysis of more than 400 subtype A and Subtype C infected individuals in the entire Protocol C cohort (Prentice et al., 2014). For each of the 30 individuals, we PCR amplified near full-length single-genome amplicons from acute infection plasma samples to define the TFV sequence and then constructed IMC using the methodology described in Deymier et al. (2014). We compared the replicative capacity (RC) of virus derived from these TFV IMCs in activated PBMCs over a 12-day period. We calculated the RC score by quantitating every 2 days the amount of reverse transcriptase activity in the culture medium, determined the area under the curve for each virus, and normalized it to that of the primary isolate MJ4 (Deymier et al., 2015). While RC scores varied over 10-fold for viruses of each subtype, we found no significant difference between the median RC scores of subtype A and C TFVs (Figure 1B; median RC scores of 1.29 vs. 1.21 respectively). Based on this limited number of TFV, the results suggest that differences in the *in vitro* replicative capacity of subtype A and C TFVs cannot explain the observed differences in clinical outcomes between the two subtypes.

**Table 1:** Acute infection subjects for IMC construction.

Protocol C ID	Days post-infection	GB accession number	Subtype	RC value
175020	46	MT942787	A1	2.58
175005	14	MT942708	A1	0.23
175038	21	JX236678	A1	0.80
175090	15	MT942927	A1	1.74
175059	17	MT942819	A1	5.10
175071	41	MT942857	A1	0.56
175092	25	MT942928	A1	0.65
175094	16	MT942955	A1	1.02
175065	14	MT942836	A1	1.34
175027	67	PQ246051	A1	1.23
175014	46	MT942748	A1	2.63
175093	15	MT942941	A1	3.29
175019	10	MT942773	A1	3.24
175010	73	MT942722	A1	0.62
305136	43	MT347680	C	2.82
305119	46	PV430297	C	0.11
305156	17	MT347681	C	0.82
305144	26	PV430298	C	2.11
235082	42	PV430296	C	0.23
305153	14	PV430300	C	2.48
235234	32	MT347679	C	2.91
235212	17	KR820325	C	0.41
305161	8	PV430299	C	3.30
235214	35	KR820323	C	0.95
235094	66	PV430295	C	1.34
235092	43	MT194496	C	3.11
235219	14	KR820366	C	0.88
235239	14	KR820421	C	1.09
235252	24	KR820449	C	0.10
235227	20	KR820393	C	3.35

### Biomarker responses after acute subtype C infections exhibit a greater increase compared to that of subtype A infections

**Figure 2:**
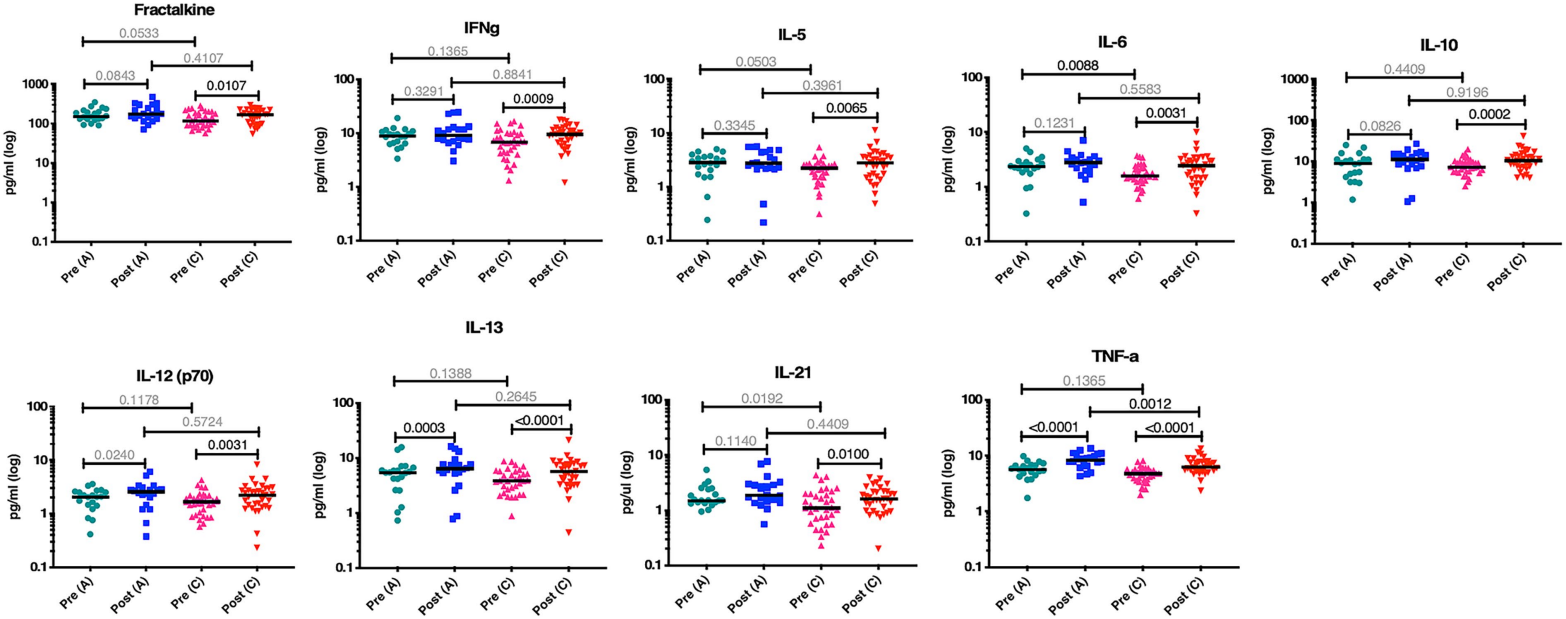
More biomarkers are significantly increased post infection in HIV-1 C compared to subtype A. Nine biomarkers are increased post infection in acute subtype C infection compared to only two in subtype A infection. Teal data points are biomarker concentrations pre-subtype A infection (*n* = 20), blue is post-subtype A infection (*n* = 20), pink is pre-subtype C infection (*n* = 34), and red is post-subtype C infection (*n* = 34). Wilcoxon matched-pairs signed rank test, two-tailed was used between pre and post levels within a subtype. Kolmogorov–Smirnov test, two-tailed was used for remaining comparisons between subtypes.

We next investigated the possible role that innate immune responses, and specifically inflammatory cytokines/chemokines, might play in the subtype differences in disease progression observed in the Protocol C cohort. To address this question, we identified a total of 20 subtype A individuals and 34 subtype C individuals for whom we had plasma samples collected prior to and shortly after the EDI (Table 2). Using a multiplex Luminex assay for 21 inflammatory cytokines and chemokines, we compared the biomarker levels in individuals pre- and post-infection with either subtype. The characteristics of the plasma samples used in this study are summarized in Table 2. For both the subtype A and subtype C individuals tested, the pre-infection samples were taken a median of 46 days before the estimated date of infection. For the post-infection samples, samples from both cohorts were collected less than 3 months after the estimated date of infection (Table 2). We observed that nine biomarkers (fractalkine, IFNγ, IL-5, IL-6, IL-10, IL-12, IL-13, IL-21, and TNFα) had significantly increased levels post-infection with subtype C HIV-1 compared to the pre-infection levels. In contrast, in subtype A infected individuals, only two biomarkers (IL13 and TNFα) had significantly increased levels post-infection (Figure 2). To account for the multiplex nature of the assay, we only considered *p* values less than or equal to 0.01 as significant. Despite the fact that subtype A infected individuals exhibited fewer significant increases in biomarker levels following infection, the absolute levels of most biomarkers approximately 1 month after infection, with the exception of TNFα, were not significantly different between individuals infected with the two subtypes. In large part this appeared to be the result of higher inflammatory biomarker levels prior to infection in Rwandan subtype A individuals. The median pre-infection biomarker level was higher in each instance, and this difference was significant for IL-6 (*p* = 0.0088) and trending for IL-21, IL-5 and fractalkine (*p* = 0.0192, 0.0503 and 0.0533 respectively).

**Table 2:** Subjects for plasma cytokine analysis.

Subtype	Number of subjects (total = 54)	Gender	Days pre-infection	Days post-infection
Subtype A	20	11 male/9 female	Median 46.5 (range 19–107)	Median 25.5 (range 11–72)
Subtype C	34	19 male/15 female	Median 46 (range 12–115)	Median 44.5 (range 14–74)

### Partial least squares analysis confirms the greater number of elevated biomarkers in subtype C immune responses

**Figure 3:**
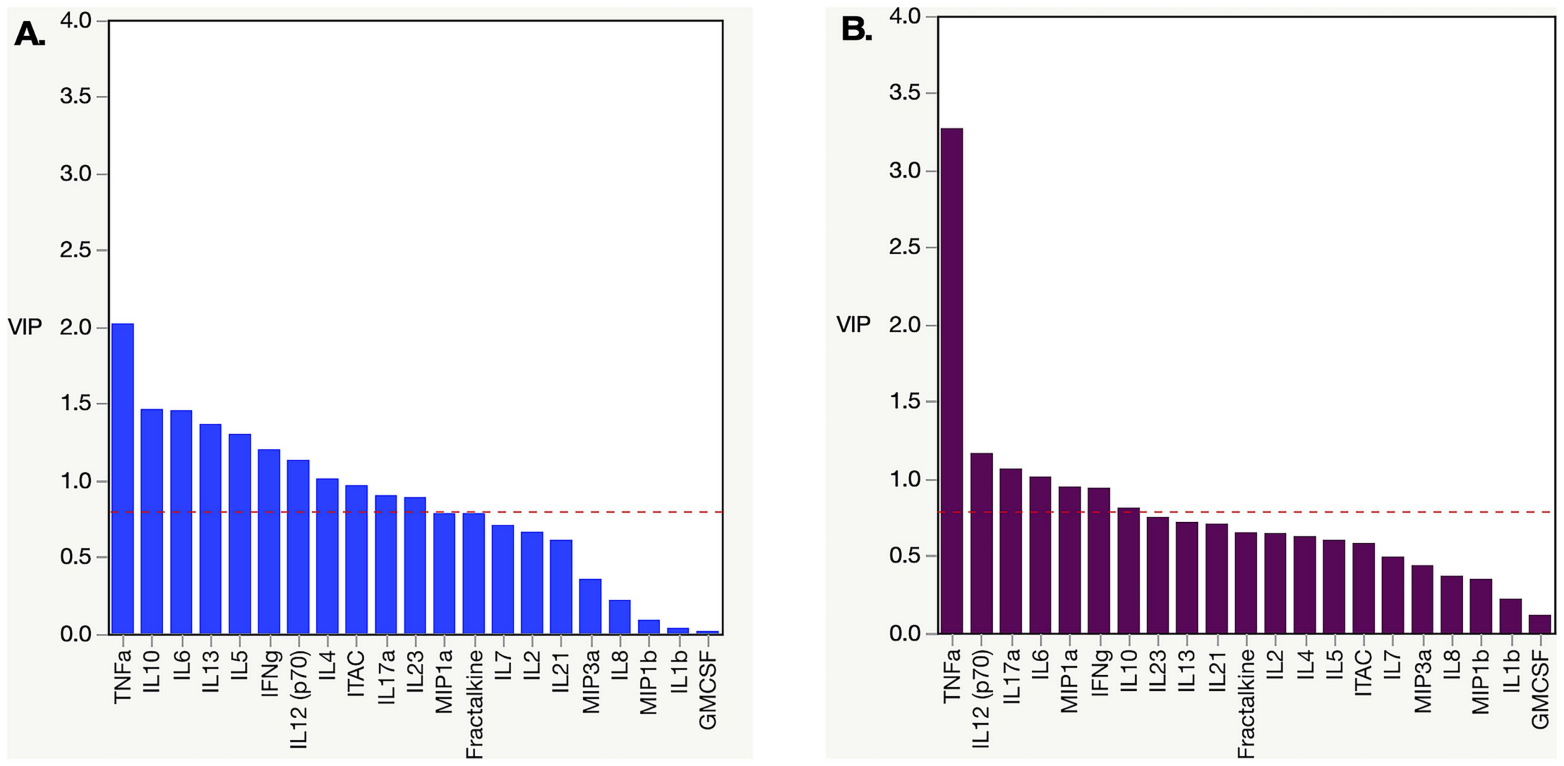
Partial least square (PLS) analysis of changing biomarker profiles pre and post infection in HIV-1 subtype C and A acute infection cohort. **(A)** PLS Analysis for Zambian subtype C cohort dataset from Figure 2. TNFα, ITAC, IFNγ, IL10, IL12, IL13, IL17α, IL23, IL4, IL5, and IL6 were all increased post infection. **(B)** PLS analysis for Rwandan subtype A cohort dataset from Figure 2. TNFα, IFNγ, IL12, IL17α, IL6, and MIP1α were all increased post infection. Analysis was done with NIPALS fit with one factor VIP (variable importance plot) threshold was set at 0.8.

When we performed partial least squares analysis on the pre-post infection cohorts for both subtypes, they were consistent with the results from our earlier univariate analyses. The goal of this multivariable analysis was to identify, in the context of frequently covarying factors, those analytes that contributed most strongly to the differences between pre- and post-infection. In subtype C, we found that the changes in the biomarker profiles post infection compared to pre-infection were due to increases in TNFα, IL-10, IL-6, IL-13, IL-5, IFNγ, IL-12, IL-4, ITAC, IL-17α, and IL-23 (Figure 3A). Changes in the subtype A biomarker profile were limited to increases in just TNFα, IL12, IL17α, IL6, MIP1α, and IFNγ (Figure 3B). For subtype A, most of the variance is due to an increase in TNFα, whereas multiple biomarkers, including elevated TNFα, contribute to the variance seen in subtype C acute infection.

### Impact of viral RC on disease trajectory and cytokine production in acutely infected HIV-1 individuals

**Figure 4:**
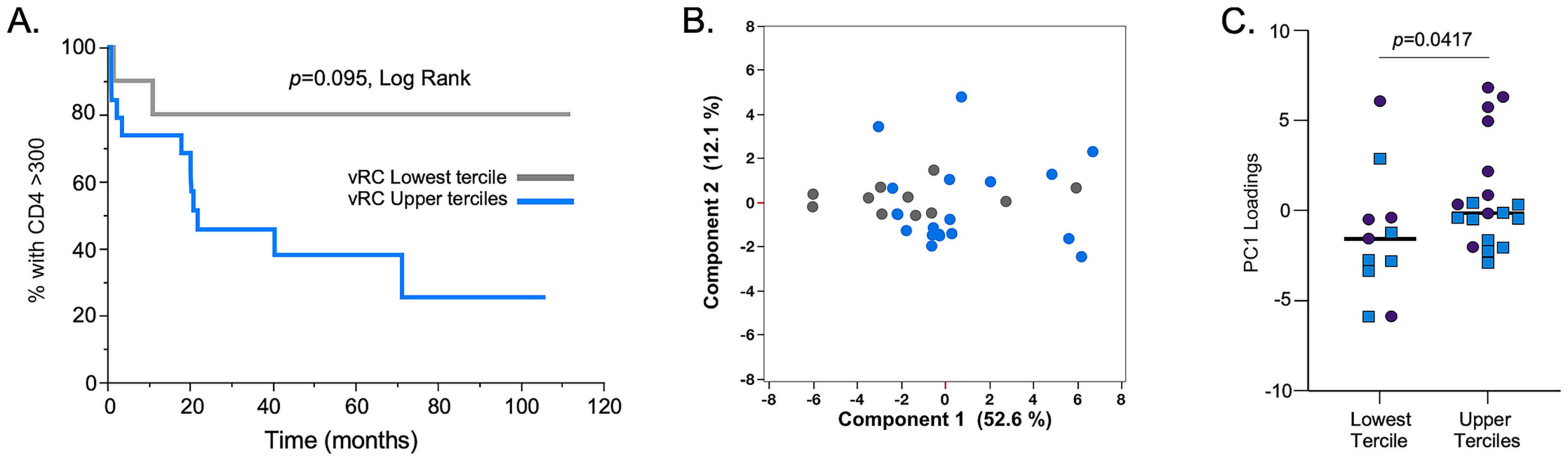
*In vitro* replication capacity of full-length TF IMCs is associated with CD4^+^ T cell loss and inflammatory cytokine profiles. **(A)** Replicative capacity assays with viruses derived from IMCs of both HIV-1 subtype A (*n* = 14) and C (*n* = 16) showed that those with viral RCs in the upper 2/3 terciles (blue line) lose CD4^+^ T cell counts at a faster rate compared to IMCs with RCs in the lowest tercile (gray line). **(B)** Principal component 1 (PC1) and principal component 2 (PC2) scores are shown in a two-dimensional scatter plot for all 30 individuals used in the RC assays. The analysis showed separation of cytokine responses based on RC terciles (lowest RC tercile, gray dots; upper 2/3 terciles), blue dots. **(C)** The lowest RC tercile showed lower PC1 loadings compared to the upper 2/3 RC terciles. Subtype C samples: blue squares; subtype A samples: purples circles. Mann–Whitney, two-tailed analysis was performed to assess significance.

While we observed no significant differences between the RCs of subtype A and C TFVs, the availability of these IMCs allowed us to reexamine the impact of RC on disease trajectory. In a previous study, we examined this through the construction of 127 *gag*-MJ4 chimeric viruses and found that low viral RC was significantly associated with individuals who exhibited slower CD4^+^ T cell decline, whereas high viral RC was associated with rapid CD4^+^ T cell loss (Claiborne et al., 2015). In the current study, despite the significantly smaller sample size, we observed similar associations between viral RC and CD4^+^ T cell decline. Viruses that had RCs in the lowest tercile of viral RC values had a slower rate of CD4^+^ T cell decline compared to viruses with RCs in the upper two terciles of RCs. Although there was clear evidence of a trend (*p* = 0.095, Log Rank), these differences were not significant, presumably because of the small numbers (Figure 4A). A Cox proportional hazards model with both RC and B*81, an HLA-I allele highly protective for CD4^+^ T cell loss, was significant for RC when the upper and lower terciles were compared (Table 3; *p* = 0.049, HR = 3.70).

**Table 3:** Cox proportional hazards model, ^a^time to CD4 <300.

Factors tested	HR	*p*-value
High vRC	3.7	0.049
B*81	9.9^−10^	0.02

HR, hazard ratio. High vRC defined as the upper two terciles of vRC values in the distribution.

a Multivariable Cox proportional hazards model with an endpoint defined as a single CD4^+^ T-cell count reading below 300. High vRC defined as the upper two terciles of vRC values in the distribution.

We also compared the levels of systemic inflammatory cytokines/chemokines of these individuals during acute infection, since our previous study showed a strong inflammatory profile associated with high viral RC. Using a principal component analysis, we again found that there was a separation of cytokine profiles between individuals that were infected by viruses in the lowest tercile of RC compared to those with RCs in the upper two terciles (Figures 4B,C). Specifically, Figure 4C shows that PC1 loadings for cytokine responses were significantly smaller for the lowest tercile RC viruses compared to upper 2/3 tercile RC viruses.

### ITAC (CXCL11) is a marker for CD4^+^ T cell decline in the combined cohort

**Figure 5:**
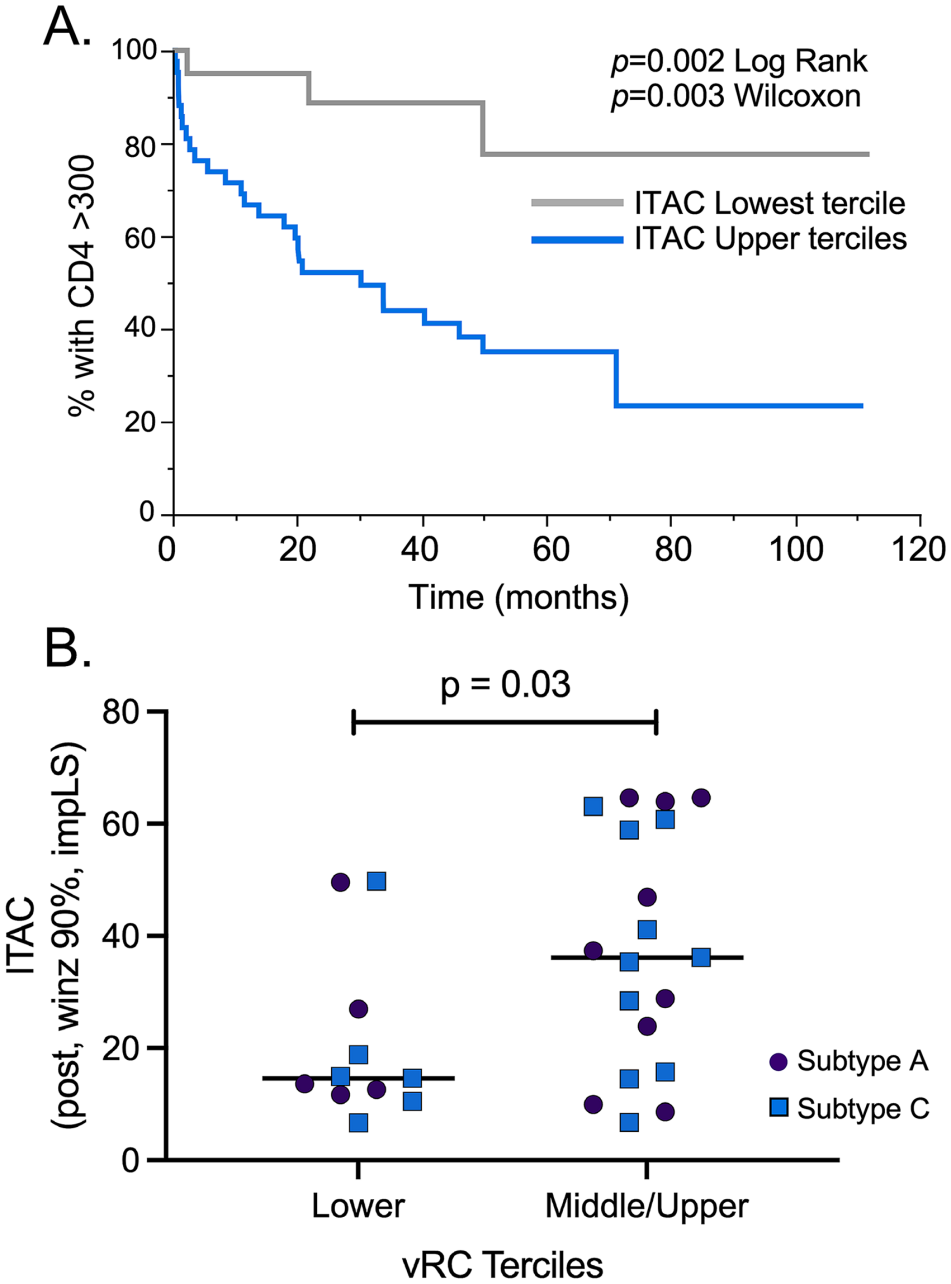
ITAC is a biomarker associated with HIV pathogenesis and higher RC. **(A)** A total of 84 post infection ITAC values were mean centered and normalized to minimize batch effects in Luminex assays. A Kaplan–Meier analysis of the combined subtype A and C cohorts showed that individuals with ITAC values in the upper 2/3 terciles (blue line) had a more rapid CD4^+^ T cell count decline compared to the low ITAC tercile (gray line). **(B)** Comparison of the lowest and the upper 2/3 terciles of RC values showed that the upper 2/3 terciles had significantly increased ITAC levels compared to vRC values in the lowest tercile. Subtype C samples: blue squares (*n* = 16); subtype A samples: purple circles (*n* = 14). Student’s *t*-test, two-tailed was used to assess significance.

To identify potential post-infection biomarkers associated with disease progression, the 84 acutely infected subtype A and C individuals included in this study were evaluated in a Kaplan–Meier analysis. All cytokine-chemokine values were mean centered and normalized to avoid any batch effect that may have existed between the different Luminex experiments. ITAC was the one analyte that stood out as being linked to disease progression; post-infection ITAC values in the upper two terciles were significantly associated with more rapid decline of CD4^+^ T cells to below 300 compared to post-infection ITAC values in the lower tercile (*p* = 0.002 Log Rank; *p* = 0.003 Wilcoxon; Figure 5A). When the risk ratios were calculated, ITAC values in the upper two terciles were associated with a greater than 5-fold risk of more rapid CD4^+^ T cell decline compared to those in the lower ITAC tercile [R.R. 5.65 (1.72–18.58), *p* = 0.004]. In addition, when the normalized ITAC values were compared within individuals for whom there were TF viruses that had RC values, RCs in the upper two terciles were significantly associated with higher ITAC values compared to individuals in the lower RC tercile (Figure 5B) linking viral phenotype to a diagnostic cytokine.

## Discussion

We have previously shown that the phenotype of the virus that initiates infection in an individual, the transmitted founder virus, can significantly impact both viral load and disease trajectory (Prince et al., 2012; Claiborne et al., 2015; Yue et al., 2015). In a previous study of more than 120 individuals newly-infected with subtype C HIV-1, we demonstrated that the replicative capacity (RC) of the infecting virus was associated both with set-point viral load and the speed of CD4^+^ T cell decline (Prince et al., 2012; Claiborne et al., 2015). A number of studies comparing the replicative capacity of viruses, chimeric in either *gag* or *env* genes, have suggested that different subtypes have different replicative capacities (Archary et al., 2010; Venner et al., 2016). In a limited study of five subtype A and five subtype D authentic molecularly cloned TFVs, we showed that subtype D TFVs replicated better than their subtype A counterparts (Baalwa et al., 2013). Because subtype A HIV-1 infected individuals exhibit similar disease characteristics as individuals infected with low vRC viruses of other subtypes, we sought to determine whether authentic transmitted founder viruses derived from acutely infected subtype A and subtype C individuals similarly differed in their replicative capacity.

In this study, where we compared a total of 30 viruses derived from molecularly cloned TFVs; we did not find a significant difference in the replicative capacity of HIV-1 subtype A and C TFVs. Based on this data, it does not appear that replicative capacity in activated CD4^+^ T cells *in vitro* can explain the differences in disease progression observed in the two subtypes. However, it should be noted that the small number of viruses analyzed from each subtype limits the strength of this conclusion and may make subtle but potentially biologically important differences harder to detect. Nevertheless, the results are consistent with the findings of Prentice et al. (2014), who showed that viral loads at 3 months after infection were similar for subtype A and C infected individuals.

When we examined some of the characteristics associated with disease progression, we saw a lack of association between RC and set point viral load (data not shown), consistent with a previous study where the association between the two factors was weak (Prince et al., 2012). In contrast, we were able to observe a clear separation of the Kaplan–Meier curves for low RC and high RC viruses and time to CD4 counts under 300, which while not significant, likely due to the small number of viruses, does support an association between RC vs. CD4 decline in this study of authentic TFV. This is consistent with findings from a previous study that examined 127 gag-MJ4 chimeric viruses and showed that low viral RC was significantly associated with slower CD4^+^ T cell decline, while high viral RC was associated with rapid CD4^+^ T cell decline (*p* = 0.002; Claiborne et al., 2015). In addition, both this previous study and our current study found that high RC viruses are linked to higher levels of inflammatory cytokines and chemokines (Claiborne et al., 2015).

Since inflammation is known to be a driver of disease progression and CD4^+^ T cell loss in HIV-1 infection (Fiebig et al., 2003; Roberts et al., 2010; Jiao et al., 2012; Shive et al., 2014; Claiborne et al., 2015), we wanted to determine whether there were differences in inflammatory biomarkers that could explain the differences in CD4^+^ T cell decline in subtype A and C infected individuals in the Protocol C cohorts (Amornkul et al., 2013). We found a greater number of biomarkers were increased as a result of HIV-1 subtype C infection compared to subtype A infections, but, while differences in biomarkers levels were observed within subtypes before and after infection, the overall levels of the biomarkers following HIV-1 acute infection were the same in the two subtypes. In addition, while subtype C infections result in the increase in multiple biomarkers post infection, subtype A infection is mostly defined by an increase of TNFα. The reduced number of cytokines that increased following subtype A HIV-1 infection was due in part to the higher levels of biomarkers observed prior to infection in these individuals. We cannot rule out the possibility that it is the magnitude of increase from uninfected levels of inflammatory cytokines/chemokines to those after infection, rather than the absolute levels themselves, that mediates the immunological dysfunction and elevated CD4^+^ T cell decline observed in subtype C.

The cause for the differences observed between HIV-1 subtype A and C infections remains unclear. One confounding aspect is that subtypes are generally linked to geographic locations, with subtype A being predominantly found in East Africa (Rwanda, Kenya and Uganda) while subtype C is strongly associated with southern African countries (South Africa and Zambia). The ethnic and genetic differences that distinguish individuals in Rwanda and Zambia could therefore influence the immune responses to these viruses. Indeed, one possible explanation is that subtype C viruses are better adapted to the immune system of their new hosts than subtype A viruses. We have previously shown that infections by subtype C TFVs that have a higher fraction of epitopes preadapted to the HLA-directed immune response of their new host result in higher viral loads and faster CD4^+^ T cell decline (Carlson et al., 2016; Monaco et al., 2016). In the study by Monaco et al. (2016), viruses from 169 subtype C heterosexual transmission pairs were sequenced and on average one-quarter of the possible HLA-linked target sites in the transmitted virus Gag proteins were already adapted to the new host’s HLA types, with transmitted preadaptation significantly reducing early immune recognition of epitopes. While the extent of immune adaptation in subtype A viruses to their hosts is currently unknown, it is possible that the ongoing viral control observed in subtype A infections reflects less adaptation to their Rwandan hosts compared to subtype C infections in Zambian individuals; ongoing studies are aimed at addressing this question.

In an unexpected finding, we were able to find an association between CD4^+^ T cell count decline and higher levels of the inflammatory chemokine ITAC when we combined the cohorts in this study. ITAC, or CXCL11, is produced by multiple immune cells (neutrophils, monocytes, and macrophages) and endothelial cells in response to IFNγ and binds to CXCR3, which is preferentially expressed on Th1 cells (Cox et al., 2008). Of the defined CXCR3 ligands, ITAC is the most potent and efficacious for chemotaxis of activated T cells and has the greatest affinity for CXCR3 as well (Petkovic et al., 2004). One study found that the presence of ITAC in an *in vitro* transwell migration model resulted in a 4–6 fold increase in the migration of T cells (Mohan et al., 2002). Another study from 2005 found that ITAC mRNA levels were upregulated in HIV-1 infected human monocyte-derived macrophages and dendritic cells. Medium from these infected cells were then found to be chemotactic for freshly isolated human CD4^+^ T cells; however, when the CD4^+^ T cells were pretreated with an anti-CXCR3 antibody (the receptor that ITAC binds to), the previously observed chemotaxis was abolished (Foley et al., 2005). This study also found that ITAC mRNA levels were upregulated within a lymph node isolated from an HIV-1 infected individual, which implicates ITAC in the recruitment of susceptible CD4^+^ T cells to HIV-1 infected lymph nodes (Foley et al., 2005). The authors of this study hypothesize that since CCR5^+^ T cells usually also express CXCR3, ITAC plays an important role to recruitment of these susceptible cells to HIV-1 infected regions or cells; this recruitment might then be able to “enhance the sequestration of T cells in infected lymphoid organs and spread of HIV-1 infection between cells” (Foley et al., 2005). Overall, this previous study is consistent with the results presented here that ITAC can be a contributor to AIDS immunopathology.

Previous studies have identified CD4^+^ T cell decline and viral replicative capacity are both markers of more rapid disease progression (Giorgi et al., 1999; Prince et al., 2012; Claiborne et al., 2015). Our results raise the possibility that high ITAC levels may be a marker for rapid disease progression. This result falls in line with our previous observations that high ITAC levels in uninfected partners were associated with HIV-1 acquisition and that the ITAC levels could be used as a predictive variable to identify individuals that would eventually seroconvert within serodiscordant couples (McInally et al., 2021). The findings of the current study show that ITAC may not only be associated with susceptibility to HIV-1 acquisition, but also disease progression in HIV-1 infected individuals. They highlight the need to further examine the role of ITAC in HIV-1 infection.

Overall, the data presented here demonstrate similar *in vitro* replicative capacities for subtype C and A transmitted founder viruses, arguing against this phenotypic trait as the basis for different pathogenic outcomes in the two subtypes. Moreover, levels of inflammatory cytokines that might drive disease progression were similar during acute infection indicating that additional studies are required to understand the mechanism underlying differences in disease progression between the two subtypes.

## Materials and methods

### Study subjects

All participants were enrolled in the Rwanda Zambia HIV Research Group (RZHRG) discordant couple cohorts in Lusaka, Zambia and Kigali, Rwanda. Subjects from both cohorts were enrolled in human subjects protocols approved by the Emory Institutional Review Board, the Rwanda National Ethics Committee and the University of Zambia Research Ethic Committee and provided written consent. When the participants enrolled in the cohort and during each visit, they were provided couples counseling and testing, and condoms to reduce transmission of HIV-1.

Infectious molecular clones (IMC) were derived from seropositive individuals a median of 19–25 days post-estimated date of infection (EDI) (Table 1). All of the subtype C individuals came from Zambia. In the Zambia cohort, the median days post-EDI was 25. All of the subtype A individuals came from Rwanda. In the Rwanda cohort, the median post-EDI was 19. For biomarker studies, all individuals had samples collected pre-EDI and within 3 months of the EDI. For the subtype A cohort, samples were taken a median of 47 days before the EDI and 26 days after the EDI. For the subtype C cohort, samples were taken a median of 46 days before the EDI and 45 days after the EDI (Table 1B). The algorithm used to determine the EDI has been previously described (Haaland et al., 2009).

### Replicative capacity assays

Generation of IMCs was as described in Shive et al. (2014) and Deymier et al. (2014). The generation of viral stocks, determination of particle infectivity, and replicative capacity assays are fully described in Deymier et al. (2015). Briefly, frozen peripheral blood mononuclear cells (PBMCs) from buffy coats were thawed and stimulated with 20 U/mL of interleukin-2 (IL-2) and 3 μg/mL of phytohemagglutinin (PHA) in R10 [Roswell Park Memorial Institute (RPMI)] 1,640 Medium supplemented with 10% defined fetal bovine serum (FBS), 1 U/mL penicillin, 1 μg/mL streptomycin, 300 μg/mL L-glutamine for 72 h at 37C. 5 × 10^5^ cells were then infected in 15 mL conical tubes by 2 h spinoculation at 2,200 rpm with an MOI of 0.05 based on the titer in triplicate in TZM-bl cells. Cells were then washed five times in RPMI, resuspended in 300 μL of R10 media and plated in a 96 well plate in triplicate. 50 μL of supernatant was then sampled every 48 h starting with a day zero time point taken after spinning the plate at 1,000 rpm for 1 min to get a baseline reverse transcriptase activity for each infection well using the radiolabeled reverse transcriptase assay.

The replication score (RC score) was determined from day 2–6 time points to measure the peak of viral replication and spread. RC score for each variant was calculated using a normalized area under the curve. The median of the replicates was background subtracted using an uninfected control included at each time point and area under the curves (AUC) were divided by the AUC for a standard lab adapted subtype C virus, MJ4, to compare across the different viruses analyzed on different days.

### Evaluation of plasma biomarkers

The plasma cytokine and chemokine levels were measured using a Milliplex Map Human High Sensitivity T Cell Panel (HSTCMAG-28SK). This kit measures the levels of 21 inflammatory cytokines and chemokines. The samples were run in duplicate. In order to eliminate batch to batch variation in the assay, all tests were carried out on the same batch of plates and approximately equal numbers of pre-infection and uninfected plasma were run on the same plate. The plates were quantified and standardized on a Bioplex 2000 in the Emory CFAR Virology Core and final concentrations were extrapolated from a standard curve and expressed in pg/mL. All plasma samples were stored at −80°C and had undergone zero or a single freeze-thaw for aliquoting prior to use.

### Data analysis

Plotting of the viral load, CD4^+^ T cell counts, replicative capacity analysis, and comparison between biomarker levels were done in Prism 9. The viral load plot was generated by plotting all the available data points for all individual where data was available. Once plotted, a linear regression analysis was performed to determine if any difference existed between the viral load kinetics between the two subtypes. Slopes were compared in Prism (version 10) to calculate a two-tailed *p*-value, testing the null hypothesis that the slopes are identical. This method is equivalent to an Analysis of Covariance (ANCOVA).

Comparison between RC scores of subtype A and C IMCs was done using a nonparametric Mann–Whitney test. For intra-subtype comparisons, we used a nonparametric Wilcoxon matched-pairs signed rank test. For inter-subtype comparison, we used a nonparametric Kolmogorov–Smirnov test, and a cut-off of *p* < 0.01 was used for significant findings to address multiple comparisons.

Partial Least Squares (PLS) analysis was performed using the JMP Pro 15 statistical package. PLS analysis had a variable importance cutoff of 0.8 and was performed with a NIPALS fit with one factor.

Kaplan–Meier survival analyses and principal component analysis (PCA) were performed with JMP Pro version 15 (SAS Institute). For survival analyses, endpoints were defined as CD4^+^ T cells counts falling below 300/mm^3^, and significance is reported using the log-rank test. Risk-ratios were calculated through proportional hazards models. For PCA, cytokine data was preprocessed to winsorize extreme high values to the 90th percentile of the data distribution, and missing values were imputed through linear regression.

## Data Availability

The datasets presented in this study can be found in online repositories. The names of the repository/repositories and accession number(s) can be found below: https://www.ncbi.nlm.nih.gov/genbank/, MT942787, MT942708, JX236678, MT942927, MT942819, MT942857, MT942928, MT942955, MT942836, PQ246051, MT942748, MT942941, MT942773, MT942722, MT347680, PV430297, MT347681, PV430298, PV430296, PV430300, MT347679, KR820325, PV430299, R820323, PV430295, MT194496, KR820366, KR820421, KR820449, KR820393.
